# How should low-threshold and primary care clinics prepare to provide long-acting injectable buprenorphine? A qualitative implementation study of patient and staff perspectives

**DOI:** 10.21203/rs.3.rs-10374464/v1

**Published:** 2026-08-06

**Authors:** Andrea Jakubowski, Isabel Lamont, Zina Huxley-Reicher, Viraj V. Patel, Benjamin Hayes, Susan Spratt, Brent Gibson, Aaron D. Fox, Alex Harocopos

**Affiliations:** Albert Einstein College of Medicine/Montefiore Medical Center; Albert Einstein College of Medicine/Montefiore Medical Center; Albert Einstein College of Medicine/Montefiore Medical Center; Albert Einstein College of Medicine/Montefiore Medical Center; Albert Einstein College of Medicine/Montefiore Medical Center; OnPoint New York City; OnPoint New York City; Albert Einstein College of Medicine/Montefiore Medical Center; New York University Grossman School of Medicine

**Keywords:** Long-acting injectable buprenorphine, Implementation science, Medications for opioid use disorder, Opioid use disorder, Primary care, Syringe services programs, Low-threshold, Harm reduction

## Abstract

**Background:**

Many patients with opioid use disorder (OUD) could benefit from long-acting injectable buprenorphine (LAIB), but it is not broadly available. This study examined the implementation of LAIB in three clinics (one primary care and two “low-threshold” clinics at syringe services programs (SSP)). Objectives were to identify implementation determinants (i.e., barriers and facilitators) at the organization, staff, and patient levels to inform development of tailored implementation strategies for each setting.

**Methods:**

Researchers conducted semi-structured, qualitative interviews with staff (N = 23) and people with OUD who had lived experience with buprenorphine (N = 15) during early LAIB implementation at one primary care and two low-threshold clinics in New York City. Meeting notes from multidisciplinary clinical implementation working groups with staff (N = 27) served as an additional data source. Qualitative data collection and subsequent rapid qualitative analysis focused on implementation determinants by CFIR domain: the Innovation (LAIB), Individuals (staff and people with OUD), Inner Setting (organizational factors), and Outer Setting (external to where implementation is taking place), and recommendations for developing implementation strategies.

**Results:**

In the Individuals domain, although staff were generally enthusiastic about implementing LAIB, they lacked comfort in counseling about LAIB, which necessitated tailored training and ongoing support. People with OUD expressed a need for detailed information about LAIB (e.g., side effects, withdrawal experiences, duration of effect) and suggested a peer messenger as an information source. Strong leadership support, clinical champions, and prior experience with sublingual buprenorphine helped overcome challenges in the Inner Setting. In the Outer Setting, the requirements to obtain LAIB from specialty pharmacies and federal regulations regarding controlled substance storage posed challenges for clinics.

**Conclusions:**

In examining early implementation of LAIB in primary care and low-threshold clinics, multi-level barriers were identified as targets for implementation strategy development, including staff training needs, pharmacy access, and regulatory requirements. Favorable staff attitudes, strong leadership support, and local clinical champions helped overcome implementation barriers. These results can guide clinics in choosing implementation strategies and strengthen readiness for successful integration of LAIB.

## 1. BACKGROUND

Buprenorphine is one of only three Food and Drug Administration (FDA)-approved medications for treating opioid use disorder (OUD) in the United States. Approved in 2002, and initially available as a sublingual tablet, subsequent formulations have included sublingual films, which combined buprenorphine and naloxone to mitigate the risk of misuse, as well as long-acting injectables (LAIB), first approved in 2017 and now available in weekly and monthly doses(1, 2). While buprenorphine's approval for use in primary care clinics has expanded access to treatment and reduced barriers associated with specialized care (3), uptake of LAIB has remained slow (4)

LAIB is safe, effective, and provides a consistent level of buprenorphine for up to a month after administration (5, 6). It is potentially superior to sublingual buprenorphine for reducing non-prescription opioid use and retaining patients in treatment (7, 8) and may better prevent non-fatal opioid overdoses than sublingual buprenorphine (9). LAIB has also shown promise for particular populations with OUD, including people leaving jails and prisons (10, 11) veterans with severe OUD (12) and those receiving care for OUD in emergency departments (13, 14). However, only a fraction (2%) of the over 9 million buprenorphine prescriptions written for Medicaid recipients in 2022 were for LAIB (4).

Currently, there is limited understanding of why LAIB has been slow to gain traction. Generally, the adoption of new medications is influenced by multiple factors, including the patient (interest and preferences), prescriber (knowledge and expertise), medication properties (safety, efficacy, cost, and administrative burden), organizational (available services and resources), and external environment (reimbursement, guidelines) (15). Given its relatively recent approval, both physicians and patients may be unfamiliar with LAIB as an OUD treatment option (16, 17), and it has been theorized that the integration of LAIB into clinical practice will be a process of gradual adaptation (17). Implementation studies that provide a roadmap for clinics to adopt LAIB may, therefore, facilitate a more rapid clinical integration of this effective medication (4).

Several studies have examined LAIB acceptability to people with OUD, with many appreciating the freedom, convenience, and sense of “normalcy” afforded by a monthly instead of daily medication, though other people with OUD report loss of social support due to reduced program attendance in the transition to the monthly injection (16, 18-23). However, there is less information about staff perceptions of LAIB and implementation barriers and facilitators. Studies from Norway(24) and the United Kingdom (U.K.)(16) have reported on practitioner and policymaker perceptions of advantages and disadvantages of LAIB, but they provide limited information for organizational implementation. In comparison to sublingual buprenorphine, LAIB cannot be self-administered; therefore, clinics need procedures to acquire, store, and administer the medication. There are likely other processes that clinics develop during LAIB implementation that could serve as a model for other institutions looking to offer LAIB. Research exploring specific barriers and facilitators to implementing LAIB in clinical practice, along with strategies to address them, can be disseminated broadly to advance the field and increase uptake.

This study aims to describe system-, organization-, and individual-level barriers and facilitators to implementing LAIB in primary care and low-threshold clinics. In doing so, it offers a roadmap to implementing LAIB treatment for OUD in these settings, including the potential challenges clinics should anticipate and strategies to address them.

## 2. METHODS

This study is part of a larger mixed-methods study of LAIB implementation in low-threshold and primary care clinics (K23DA052627). This manuscript presents qualitative data collected during the early implementation phase of the study (November 2023- April 2024). Early implementation efforts consisted of convening working groups to establish clinical protocols, workflows, and identify and address implementation barriers as they arose. Data are included from multiple sources, including individual interviews and focus groups with staff and people with OUD, and meeting minutes from working groups. Findings represent early LAIB implementation barriers and facilitators, and, where relevant, strategies to address barriers. The study was approved by the Albert Einstein College of Medicine Institutional Review Board (IRB #2022–14213) and funded by the National Institute of Drug Abuse award K23DA052627.

### 2.1 Setting

A primary care clinic and two SSP-based low-threshold clinics were study sites. The primary care clinic is the Montefiore Comprehensive Health Care Center, a federally qualified health center in the South Bronx serving a low-income neighborhood with a majority Black and Latine population and among the highest overdose rates in New York City (25). At the beginning of the implementation period, there were 17 general internist physicians prescribing buprenorphine at the clinic. The two low-threshold clinics were part of OnPoint NYC, the largest SSP in New York City, which offers a variety of services, including safer-use supplies, monitored consumption spaces, case management, mental health services, and co-located medical clinics. The majority of OnPoint NYC participants are Latine or non-Latine Black and unstably housed (26). The SSP has two brick-and-mortar locations, each with its own low-threshold medical clinic staffed by three physicians, one RN, and a program manager. Both the primary care and low-threshold clinics had well-established buprenorphine programs (27, 28) and rotating addiction medicine fellows providing care, typically one day per week.

The two different clinic types each had its own advantages for implementing LAIB. The primary care clinic had existing infrastructure for administering long-acting injectable medications. The low-threshold clinics had a high volume of patients with OUD, including those with challenging life circumstances who could benefit from the consistent medication levels provided by LAIB and no requirement to safeguard the medication.

### 2.2 Implementation process

**Figure 1:**
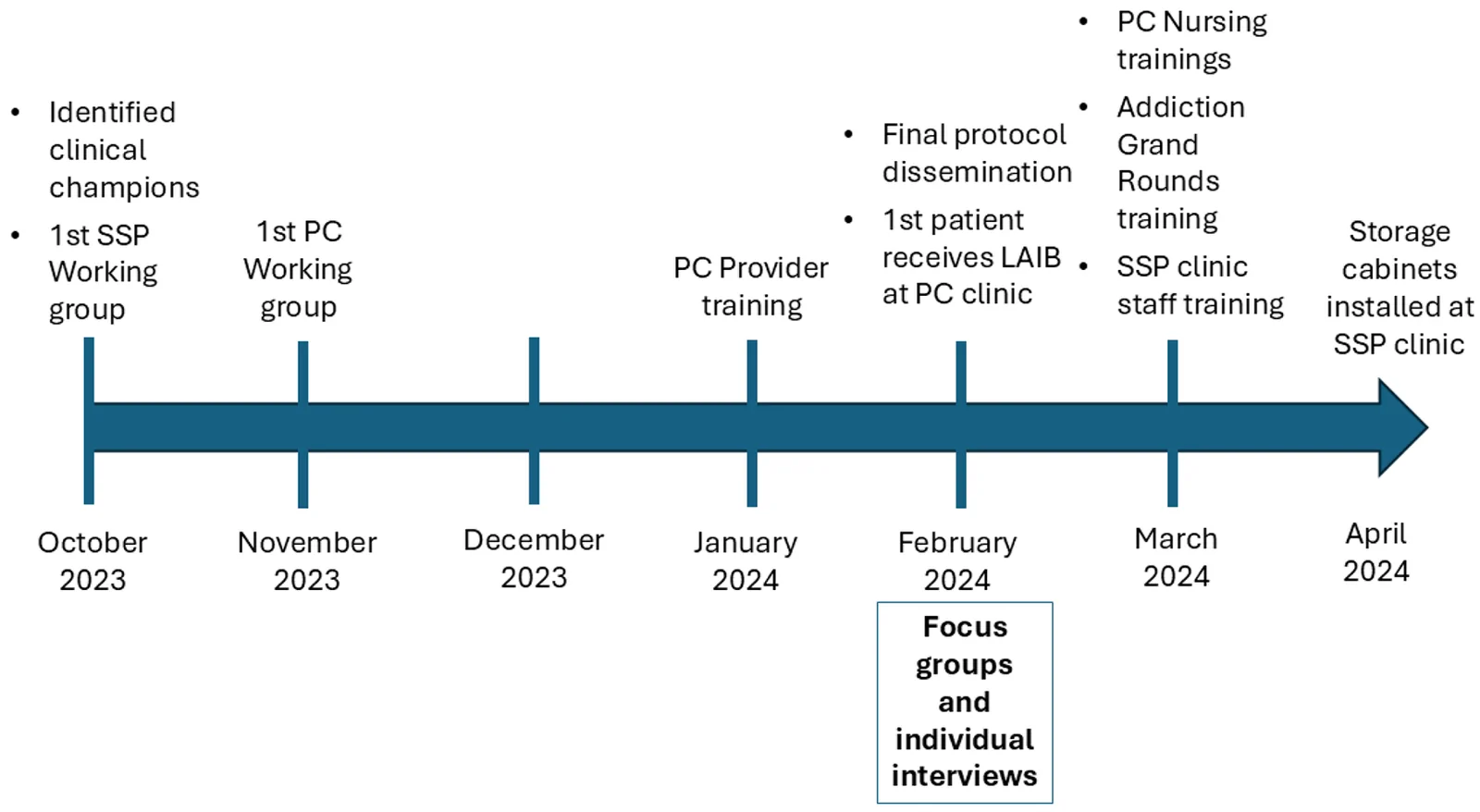
Timeline of implementation activities and data collection during the early implementation period

Implementation activities and their timing relative to data collection are shown in Figure 1. Several implementation strategies were planned at the outset of the project, and others were developed with working group feedback during implementation to address barriers as they arose. Planned implementation strategies led by the principal investigator (PI) during the early implementation phase were based on evidence-based approaches described in the Expert Recommendations for Implementing Change (ERIC) taxonomy (29). These included: identifying a clinical champion, convening multidisciplinary clinical implementation working groups (hereafter called “clinical working groups”) focused on developing and refining clinical workflows, and facilitation(30) (30) (See Box 1).

Clinical working groups were used as a community engagement tool to ensure participation from staff in a variety of roles and to ensure that implementation activities were useful to the staff providing LAIB services in each setting. Feedback from clinical working groups, focus groups, and individual interviews led to the development of new approaches and modifications to existing ones as needed. Training slide decks, the final clinical protocol, and patient-facing handouts are included in Supplementary Files 1–5.

### 2.3 Participants

Participants in the study included staff and people with OUD. Staff included physicians, nurses, front desk staff, clinic administrators, and, at the SSP sites, harm reduction staff in various roles. People with OUD were eligible if they had received services at clinic sites and had current or past experience with sublingual buprenorphine. Those not currently receiving buprenorphine were included to uncover barriers to sublingual buprenorphine or potential concerns about buprenorphine that would be important to address in patient-facing LAIB implementation strategies. People with OUD currently receiving buprenorphine, by contrast, would likely have different concerns and require different information to inform their decision to switch from sublingual buprenorphine to LAIB.

### 2.4 Data sources

Qualitative interviews (individual interviews and focus groups) and detailed meeting minutes from the clinical working groups were data sources. Qualitative interviews and focus groups with people with OUD and staff were conducted to characterize early barriers and facilitators to LAIB implementation, with the aim of informing the development and refinement of our approach. Collecting data after the project began allowed focus groups to discuss experienced challenges rather than theoretical concerns.

All participants provided informed consent. Staff participating in focus groups at the SSP were compensated with $50 gift cards (for a 1.5-hour time commitment during the workday). At the primary care clinic, clinical policies prohibited staff from receiving an honorarium for study participation; however, all study activities were conducted during participants’ usual working hours. All participants with OUD were provided with $35 gift cards for their participation (for a 1-hour time commitment).

#### 2.4.1 Staff focus groups

Researchers conducted three focus groups with a total of 23 staff across the two settings at a single point in time (Month 4 of implementation; See Fig. 1). At the primary care clinic, separate focus groups included physicians (n = 6) and other staff (front desk staff, administrators, and nurses, n = 7). At the low-threshold clinics, one focus group included 10 non-clinical staff members (three case management/mental health staff; two buprenorphine case counselors; four harm reduction specialists; and one drop-in center manager). Due to the small number of clinicians at the low-threshold clinic, data regarding implementation determinants were collected during the multidisciplinary clinical working group meetings instead of focus groups (see Section 2.4.3).

#### 2.4.2 Focus groups and interviews with people with OUD

A total of 15 people with OUD participated in focus groups or individual interviews. One focus group with people with OUD at a low-threshold clinic included participants who had buprenorphine experience but were not currently receiving buprenorphine treatment (n = 7), and another at the primary care clinic included people with OUD who were currently receiving buprenorphine treatment (n = 4). To ensure a diversity of viewpoints within each site, researchers conducted individual interviews with two primary care clinic patients with buprenorphine experience who were not currently receiving medication and two patients who were currently receiving buprenorphine treatment at the low-threshold clinics.

Focus groups and individual interviews with people with OUD and staff were conducted by the PI (AJ) and the study coordinator (IL), both of whom have extensive backgrounds in qualitative research. Interviews were audio-recorded and transcribed. Interviewers completed detailed post-interview and post-focus group memos to synthesize key takeaways and maintain a record of contextual and nonverbal descriptions pertinent to the analysis.

#### 2.4.3 Clinical working groups

PI-led clinical working groups were convened at the outset of the project and comprised of nursing, front desk staff, administrators, and physicians. A total of 12 work group meetings were held biweekly, each lasting 30 to 45 minutes, with 27 staff members participating in one or more meetings. Primary care clinical working group members included nine physicians, three clinic administrators, one registered nurse (RN), and one clinical pharmacist. The low-threshold clinic working group included four physicians, three RNs, two patient navigators, one clinic manager, two patient care navigators, and one psychiatric-mental health nurse practitioner. A study coordinator took detailed notes during meetings, which were then reviewed and edited by the PI for accuracy.

### 2.5 Measures

#### 2.5.1 Staff interviews

Interview guide development was based on the CFIR, including the major CFIR domains and selected subdomains considered most relevant to LAIB implementation based on prior literature (31-33). Topics included: the relative advantage of LAIB compared to sublingual buprenorphine or other MOUD; available resources; work infrastructure and staff capability; access to knowledge; information and motivation to support LAIB implementation; organizational culture; the relative priority of LAIB implementation compared to other clinical initiatives; perceptions of people with OUD needs; and practical strategies for LAIB implementation in both primary care and low-threshold settings. All interview guides are included in Supplementary File 6.

#### 2.5.2 Interview with people with OUD

Development of the interview guide was informed by prior literature on perceptions of LAIB among people with OUD (34, 35). Domains included: concerns regarding LAIB, information they would want to have when considering LAIB treatment, and how they would prefer that information be provided. Interviews also solicited feedback on a draft of an LAIB patient handout (final drafts included as Supplementary Files 4–5). Questions for SSP participants included whether LAIB fit with the harm reduction mission of the low-threshold setting.

### 2.6 Analyses

Rapid qualitative analytic methods were used to efficiently synthesize early data on implementation barriers and facilitators to inform the timely development of LAIB implementation strategies (36).

Researchers created interview analysis summary templates organized by interview domain. Analysts used the template to extract data from interview transcripts, summarizing question responses, extracting illustrative quotes, and noting implications for implementation strategy development/refinement. Staff summary templates also included a column for the CFIR domain (Innovation, Individuals, Inner Setting, and Outer Setting) and subdomain coding (an example summary template is included in Supplementary File 7). One to two analysts (AJ and IL) reviewed interview transcripts and completed interview summary templates (two coders reviewed data items that were longer or more in-depth). Disagreements between analysts were reconciled during in-person meetings. Staff focus group templates were not aggregated into a matrix for analysis because interview questions varied by staff role, so aggregation was not appropriate.

Focus groups and individual interviews conducted with people with OUD were extracted into summary templates as previously described, but without categorizing the data by CFIR domain, given that the CFIR only has a single domain explicitly addressing Innovation Recipients (i.e., patients or service recipients). People with OUD templates were aggregated into a matrix for analysis.

Clinical working group meeting minutes were extracted by the study coordinator directly into a matrix organized by major CFIR domain. The matrix included a column for implications for implementation strategy development/refinement. The PI then reviewed and edited all matrices.

For the final analytic step, all summary templates, working group and patient matrices, and post-interview memos were reviewed and synthesized into a summary of the significant findings.

## 3. RESULTS

Early implementation determinants are highlighted by CFIR domain—Innovation, Individuals (Innovation Deliverers and Recipients), Inner Setting, and Outer Setting—and highlight thematically relevant subdomains. Each section also presents programmatic adaptations when relevant. Table 1 summarizes barriers and facilitators by CFIR domain and notes how they were addressed.

### 3.1 CFIR Innovation domain

The “Innovation” was long-acting injectable buprenorphine (LAIB). Early clinical working group discussions focused on selecting a formulation (Brixadi^®^, Sublocade^®^, or both). Initially, sites focused on Brixadi^®^ to avoid overwhelming physicians and because of practical advantages: no refrigeration requirement, multiple injection sites, smaller syringe gauge and injection volume, and availability in weekly and monthly formulations.

Staff at both sites perceived advantages of LAIB over sublingual buprenorphine or other MOUD (CFIR: Relative advantage). Clinical working groups highlighted that fentanyl impeded sublingual buprenorphine initiation and appreciated a new option. Clinical staff at the low-threshold clinic thought LAIB had potential benefits for specific scenarios: when sublingual buprenorphine was frequently lost or stolen, for patients not taking medicine as prescribed, or if patients were not progressing toward their goal of reducing or stopping non-prescribed opioid use.

Harm reduction staff at SSP sites, who were less familiar with buprenorphine than clinical staff, were also generally enthusiastic about LAIB’s potential benefits. They highlighted advantages such as receiving a monthly injection instead of attending a methadone program daily, the fit of LAIB for patients with a “recovery” mindset, and its usefulness for patients who avoided sublingual buprenorphine because of its taste.

“I feel like it doesn't consume as much time … [as] going to say a methadone clinic every day… or taking a strip every certain time of day, or I feel like it gives you more time to move around and not have to worry too much about getting medicated… you take that once a month and that's it. You're good. So, I feel like that's a very good way of starting your recovery process. If that's what they're looking for, it's beneficial.”

(SSP Focus group participant #4)

However, harm reduction staff also proposed that transitioning to LAIB could be challenging for people using non-prescribed opioids to numb emotional and physical pain. Their concern was that some patients could experience intense emotions that previously had been suppressed or managed by the opioids they were using, and suggested that patients starting on LAIB might benefit from additional emotional support:

“And you got people that been injecting heroin 10, 15 years and they might want to try an injection [LAIB]. But like I said, counseling is a big thing, making him feel comfortable because the person might be on bupe, he got the injections. But seven days later, his feelings and his emotions are starting to kick in and he doesn't know how to deal with that because he's been injecting drugs in himself for 10, 15 years. And I think emotional support group is a big, big, big thing.”

(SSP Staff Focus group participant #5)

### 3.2 CFIR Individuals domain

#### 3.2.1 Innovation deliverers (Staff)

Staff at the primary care and low-threshold clinics were highly motivated to implement LAIB to increase patient choice, but they lacked preparedness (CFIR domains: Motivation; Capability). Although physicians received introductory lectures on LAIB, primary care clinic physicians lacked confidence in counseling patients about what to expect from the medication. Physicians at both clinics valued the study protocol and EHR smart phrases with embedded prescribing instructions, describing them as clear and useful, but insufficient to fully address confidence gaps.

“I feel moderately confident in counseling [patients]. But…-- as an experienced buprenorphine prescriber, there's still, I think questions about patient experience that I wouldn't feel as comfortable telling patients about.”

(primary care physician #3)

“Do I feel like I really understand it well enough to actually provide like patient-centered counseling? Probably not yet. But I feel like I could introduce the topic, garner some interest and then sort of get some help if the patient's considering strongly”

(primary care physician #4)

While low-threshold clinic nursing staff were already familiar with LAIB and eager to offer it, primary care clinic nurses were largely unfamiliar with the medication and initially concerned that their licenses did not permit them to administer it. Primary care nurses also felt inadequately trained and expressed a preference that they first observe an LAIB administration and then receive supervised practice before administering LAIB independently. They additionally requested education on LAIB to help address patient questions during injections, including common side effects.

“As a nurse, it's not just about giving the medicine… Even though the doctor will speak to the patient. .. as a nurse, you have to understand the medication and how it works”

(primary care nurse #6)

To address lack of preparation and increase comfort levels among primary care nurses, modifications were made to the initial nurse training plan. Additions included a didactic session on LAIB with videos demonstrating injection technique, hands-on practice with addiction medicine fellows, and a session led by nursing leadership on the institution’s narcotics policy, including proper storage, administration, and documentation of LAIB.

At the low-threshold sites, harm reduction staff reported lacking confidence talking to participants about buprenorphine in general, much less LAIB (CFIR domain: Capability). They also reported a lack of resources to counsel patients about their MOUD options.

“I think I've never had all of that information to be able to answer [participant] questions. I would be comfortable doing it. It's just a matter of having that information.”

(SSP staff focus group participant #2)

“If we don't have information, there's nothing we can do because if [a participant] ask[s] a question about what it does or where it comes from and you can't answer it, they're going to disregard you. They're never going to ask you again. But if you have the right information and how the medication is going to help them and stuff, they'll think about it. Because there's a time where they can't get no money and they're sick. So that might be the point that they'd be like, yo, I want to get on the bupe.”

(SSP staff focus group participant #5)

“They want to know, am I still going to be able to get high on this? How often do I have to take it? What am I going to feel like? What's it going to be like trying to get off of this? So really like hammering in on those points that we know our participants want to know and are going to ask, but we may not necessarily know because that's not the stuff that they tell you in a research study.”

(SSP staff focus group participant #1)

### 3.2.2 Innovation recipients (People with OUD)

Participants emphasized the need for detailed information on the LAIB initiation process, including how it might affect withdrawal, how they would feel while taking it, and what discontinuation would entail. They primarily sought reassurance that LAIB would reliably “hold them” for an entire month with respect to withdrawal and cravings. Participants also wanted thorough information on side effects, interactions with other medications (e.g., HIV or mental health treatments), implications for pregnancy or infertility, and comparisons between LAIB and sublingual buprenorphine.

“Will it hold for the 30 days?…If you're three weeks in and you're feeling an urge and you call the doctor and they say, well, sorry, you got to wait until the 18th and it's the 10th, you're gonna use. You're gonna go out and use and to take that type of risk, in my situation, it's not doable.” Person with opioid use disorder currently receiving buprenorphine at primary care site

(#3)

“Side effects, yeah. I wanna know if somebody went through that process, if they had that side effect, but after down the line of being on it for a minute… Being on it for a year or two, taking the shot, if there's side effects in the long run.” Person with opioid use disorder not currently receiving buprenorphine at low-threshold site

(#3)

People with OUD expressed that they were not necessarily going to take the word of a doctor about LAIB and would prefer to hear from a peer, who had received LAIB, about their experience.

“I would have to talk to somebody that got it already. Somebody that I know, that I came across. I would like to ask them what's your experience.” Person with OUD not currently receiving buprenorphine at low-threshold site

(#4)

In response to questions about discussing LAIB in a harm reduction setting, people with OUD unanimously reported that it would not conflict with the program’s mission. People with OUD believed that staff providing information about LAIB would be perceived as supporting multiple options. They agreed that not all SSP participants would be interested in LAIB or stopping non-prescribed opioid use. Nonetheless, people with OUD discussed the value of having conversations about LAIB, which could pique participants' interest, and of having LAIB readily available when participants were ready to make a change.

“No, I don't think they'd be put off. It's a choice like either you want to …or you don't, same way is the choice to come here and get a high. .. everything falls back to the individual.” Person with opioid use disorder currently receiving buprenorphine at low-threshold site

(#1)

### 3.3 CFIR Inner Setting Domain

Within the Inner Setting, LAIB storage initially posed a challenge (CFIR subdomain: Materials and Equipment). As a Schedule III controlled substance, LAIB must be stored in accordance with federal and state regulations. At first, the primary care site relied on its co-located 340B pharmacy, which securely stored its supply as a courtesy, while the low-threshold clinics lacked compliant storage. Ultimately, both the low-threshold and primary care clinics purchased narcotics cabinets and lockboxes to meet controlled-substance requirements. New workflows were also created to receive LAIB by mail and to maintain logs of medication receipt, administration, and disposal.

Staff in both settings also lacked capacity to monitor when patients were due for their next injection, remind patients about injection appointments, and coordinate medication delivery with appointments (CFIR subdomain: Work Infrastructure). At the low-threshold clinics, the full-time RN reminded patients about injections, and physicians documented LAIB administrations and the date of the next injection in a spreadsheet shared via group email. At the primary care clinic, an RN champion assumed responsibility for tracking and reminding patients about injections but found this burdensome in addition to their usual responsibilities. However, once workflows and team communications were streamlined, the RNs’ workload related to this initiative became more manageable.

Additionally, staff experienced competing priorities in their clinical workflow. For example, physicians in both settings reported that during a busy clinical session, it was difficult to remember to introduce LAIB to patients, let alone adequately counsel and administer the medication (CFIR subdomain: Relative Priority). Physicians at the primary care clinic noted that many of their stable sublingual buprenorphine patients had other medical issues that were currently more acute than their OUD, deprioritizing discussions about LAIB.

“The reason I didn't bring it up was because the opioid use disorder became like the, fourth most important problem on the problem list and he was doing pretty stable with it [sublingual buprenorphine] and was fine… I'm not going to bring it up when there's five other things to talk about.”

(primary care physician #2)

Staff raised several issues related to the CFIR subdomain Work Infrastructure. Physicians lacked time to talk to their patients about LAIB, but they were reluctant to have other staff, such as licensed practical nurses (LPNs), introduce what they thought was a complex topic. Physicians also reported a lack of time to coordinate with specialty pharmacies regarding their patients’ insurance coverage, as well as ensuring medications were delivered in time for a patient’s injection appointment. Finally, clinic administrators were concerned about the potential for communication and workflow breakdowns, which could lead to patient dissatisfaction if patients expected to receive LAIB on a particular day, but no trained physician was available to administer it. As a result, some primary care staff advocated for a gradual rollout in which LAIB would initially be offered only one day per week, when the addiction medicine fellow and RN champion were available to assist with injections.

Several factors facilitated implementation within the Inner Setting. Addiction medicine fellows acting as clinical champions at both the primary care and low-threshold clinics were central to these efforts. Fellows provided coaching and consultation to other physicians and guided clinical working groups in developing protocols (CFIR subdomain: Access to Knowledge and Information). At the primary care clinic, physicians valued the option to refer patients to an addiction medicine expert when they lacked confidence in counseling, ordering, or administering LAIB. Having an addiction medicine fellow onsite during the first few LAIB initiations was also viewed as highly supportive.

Another key inner-setting facilitator was strong leadership support for LAIB implementation. Leadership at both the low-threshold and primary care clinics helped convene clinical working groups and, at the low-threshold sites, encouraged participation from harm reduction staff who did not work in the clinic. This approach enabled community involvement in planning and allowed implementation strategies to be tailored to each setting. Leadership at both clinics also supported the purchase and installation of secure LAIB storage, and primary care leadership collaborated with academic medical center nursing leaders to ensure regulatory compliance.

Both settings also had organizational cultures strongly supportive of buprenorphine services (CFIR subdomain: Culture). Primary care leadership described the practice culture as broadly welcoming of buprenorphine treatment, while SSP staff were enthusiastic about providing these services at the low-threshold clinic. The harm reduction culture, in particular, embraced new innovations readily, which may have accelerated implementation.

“people in the practice have embraced [buprenorphine] quite a bit over the last 20 years.”

(primary care physician #6)

“I think it's amazing that we have [buprenorphine] in house because me as the navigator, I'm able to educate people on it.”

SSP Staff focus group participant #7)

### 3.4 CFIR Outer Setting Domain

The greatest challenge within the Outer Setting was working with specialty pharmacies. Because LAIB is a controlled substance and must be administered by a healthcare practitioner, it can only be obtained through a specialty pharmacy or purchased directly from the manufacturer. In this project, physicians prescribed LAIB to a specialty pharmacy, necessitating new workflows and physician training. This process also caused delays, as the medication had to be ordered and shipped to clinics before administration. Insurance issues added further barriers. Many patients at the low-threshold clinic were eligible for New York State (NYS) Medicaid but had lapsed coverage, requiring lengthy calls to reactivate insurance. At the primary care clinic, patients with dual Medicare/Medicaid coverage or private insurance often needed prior authorizations, leading physicians to rely on addiction medicine fellows for support with prior authorizations and identifying in-network specialty pharmacies. These challenges were partially mitigated by NYS Medicaid’s coverage of all buprenorphine formulations, including supplemental sublingual buprenorphine for patients receiving LAIB.

A final barrier was the requirement that LAIB be shipped only to the address listed on a physician’s DEA license. This created difficulties when physicians practiced at multiple sites but had a different registered address. Physicians were instructed to update their DEA addresses; however, when prescribing at additional sites, they often relied on another physician with the correct registered address to order LAIB for their patients.

## 4. DISCUSSION

In this study, we described barriers and facilitators to implementing LAIB for staff and people with OUD, as well as within and external to the organization. We found that, although implementing LAIB posed an organizational challenge, strong leadership support, supportive organizational culture, and access to addiction medicine experts helped overcome early implementation hurdles. Staff at both organizations highlighted the need for more training and/or materials tailored to their roles. People with OUD preferred to learn about LAIB from a peer messenger and wanted detailed information about the withdrawal experience and potential side effects of LAIB. Staff and people with OUD both emphasized the importance of providing additional psychosocial support to patients initiating LAIB. Findings have implications at the individual (staff and people with OUD), organizational (i.e., CFIR Inner Setting), and external levels (i.e., CFIR Outer Setting), which are presented below.

At the staff level, it was notable that addiction medicine fellows were implementation facilitators at both sites, serving as LAIB champions. Both sites in this study had access to addiction medicine fellows, which limits inferences about how implementation would have progressed without their involvement as clinical champions. Many programs implementing LAIB will not have local addiction expertise and may require additional support, especially given the lack of confidence focus group participants reported despite receiving introductory training. Using clinical champions is an evidence-based implementation strategy (29), therefore, primary care programs could provide additional training to a non-addiction medicine specialists to provide consultation, coaching, and additional LAIB implementation support to other physicians in the practice. Practices without addiction expertise could also leverage national programs such as the Extension for Community Healthcare Outcomes (ECHO) program, Primary Care Support Services (PCSS) program and publicly available LAIB implementation resources (37-39). City and state health departments could also provide technical assistance and support to clinics developing LAIB programs.

Regarding the needs of people with OUD, an important concern raised by SSP staff and people with OUD was introducing a long-acting opioid blockade when patients may not have other coping mechanisms. Other research has also highlighted this concern. A study on the implementation of LAIB in a group of sex workers accessing care on a mobile van found that some patients experienced worsening post-traumatic stress disorder symptoms when starting LAIB (40). Participants in other studies have described that reducing program contact after LAIB initiation disrupted their routines and also reduced social support; they emphasized the importance of providing additional psychosocial support to patients receiving LAIB (16, 20, 22, 23, 34). Offering regular check-ins and support to all patients receiving LAIB, and linkage to mental health services for those with underlying mental health problems, may promote patient wellness and help patients stay engaged in LAIB services.

The results also reinforce patients’ information preferences (content and format). Findings were very similar to those from a qualitative study conducted in the U.K. that elicited patient LAIB information preferences, including information about pharmacology, effectiveness, safety and side effects, administration and dosing, and experience of reducing and ending treatment (35). Participants have voiced concerns about LAIB “holding” them for the entire injection dosing interval (18, 41). Finally, findings from this study suggest that people with OUD would benefit from hearing from other people with direct experience of LAIB (35, 40)

At the organization level, both sites reported strong staff support, likely due to their established and well-integrated sublingual buprenorphine programs. While LAIB was a new, more complex mode of delivering buprenorphine, it was not perceived as a drastic departure from existing services. At the low-threshold sites, neither staff nor participants expressed concern that LAIB would conflict with the organization’s harm-reduction orientation. This may be a result of implementation efforts being conducted in a community-engaged manner, as well as the existing longstanding and trusting relationship between clinic staff and SSP participants. Were there a coercive element to LAIB provision, as reported by participants in another study (20) it would be unlikely to gain support from SSP staff.

Findings also inform potential challenges to scaling LAIB nationwide. While New York State Medicaid does not require prior authorizations for LAIB, data from 2022–2023 showed that approximately one in five Medicaid managed care organizations (MCOs) across 39 states and DC did require prior authorizations for LAIB (42). Prior authorizations are a burden for physicians and create barriers for patient access to lifesaving medication (43). Moreover, some insurance plans do not cover LAIB at all. Data from 2017–2021 showed that only 46% of commercial insurance plans and 19% of Medicare Advantage plans covered LAIB (44). As clinicians are learning about LAIB, and clinics are developing new workflows for administration, additional administrative burdens could threaten successful implementation.

A strength of this study was the use of a community-engaged approach to guide implementation. Clinical working groups, staff not directly involved in implementation, as well as people with OUD, provided different perspectives and contributed directly to the development and tailoring of implementation strategies. For example, feedback informed patient-facing handout to address questions and concerns raised by patients during focus groups and interviews (handout included in Supplementary Files 4–5). In the mid-implementation phase, clinic staff and researchers iteratively developed tailored materials and training on LAIB, workshopping them along the way (to be presented in a future manuscript). Researchers also developed formal training for nurses in response to feedback received on their lack of preparedness to implement LAIB (included in Supplementary File 2–3).

The importance of community engagement in implementation practice and research cannot be overstated. The CFIR has been updated to better capture the implementation process, highlighting the importance of teaming, assessing needs, context, engaging innovation deliverers and recipients, and tailoring strategies to fit context (45). Cooper et al., underscored the benefits of community engagement in implementation science research, including engaging local knowledge and expertise, promoting authentic relationships, and building community and researcher capacity (46).

Study limitations include the use of rapid qualitative methods, which precluded application of a comprehensive CFIR codebook, so some implementation determinants may be underrepresented in results. Additionally, researchers conducting the qualitative interviews were well known to staff and were also leading LAIB implementation efforts, so participants may have tempered any reservations about LAIB or its implementation. However, steps were taken to minimize social desirability bias, including explicitly assuring staff of interview confidentiality and lack of repercussions for expressing negative views toward LAIB or implementation activities. Staff and people with OUD who participated in interviews and multidisciplinary working groups may not be representative of staff and people with OUD in general, potentially biasing findings. This study was conducted in just three urban clinics, so findings are less generalizable to rural areas. Despite its limitations, study findings can help other programs implementing similar initiatives.

## 5. CONCLUSIONS

In conclusion, while LAIB implementation had a degree of complexity and required clinic adaptation, barriers were overcome with multidisciplinary collaboration, leadership support, and favorable organizational cultures in the primary care and low-threshold clinics. Eliciting the perspectives of people with OUD early in the implementation process also contributed to tailoring patient-facing aspects of LAIB implementation. While other primary care and low-threshold clinics will need to adapt findings to their local context, the barriers to and facilitators of LAIB implementation reported here can guide development of LAIB programs to improve access to this effective medication.

## Supplementary Material

This is a list of supplementary files associated with this preprint. Click to download.

- Supp1Clinicalprotocol.pdf
- Supp2Providertraining.pdf
- Supp3Nursingtraining.pdf
- Supp4PatienthandoutsEnglish.pdf
- Supp5PatienthandoutsSpanish.pdf
- Supp6Qualitativeinterviewguides.pdf
- Supp7SummaryTemplateExample.pdf

## Figures and Tables

**Table 1.** Barriers and Facilitators by CFIR domain

*Individuals (Innovation Deliverers)*		
Barriers	How addressed	
Staff preparedness	Provision of tailored trainings, protocols, EHR smart phrases	
	Clinical champions	
	Practice facilitation	
Nursing concerns about staff roles	Leadership assurances, formal training on institutional narcotics policy, hands on training in LAIB administration	
Facilitators	How leveraged	
High motivation to implement LAIB	Participation in clinical working groups	
	Attendance at LAIB trainings	
	Working closely with local champions	
Inner Setting		
Barriers	How addressed	
Storage (low-threshold clinic)	Purchasing wall-mounted narcotics cabinets	
	Relationship with on-site pharmacy with narcotics storage capability	
Medication delivery	Establishing relationship with helpful specialty pharmacy	
	Establishing clinic workflows	
	Instruction to physicians in DEA address change, coordination among physicians when DEA address change not feasible	
Limited physician time	Referral to addiction medicine fellows	
	Protocols and EHR smart phrases	
Lack of support staff	Extending roles of existing staff	
Facilitators	How leveraged	
Addiction Medicine Fellows	Clinical champions	
	Provide 1:1 coaching and support to physicians	
	Provide training in injection administration to nurses	
	Assist with medication ordering	
	Option of sending patients to see fellows for counseling, medication ordering, and medication administration	
Leadership support	Support for convening clinical working groups, purchasing compliant storage, ensuring regulatory compliance	
Long-standing buprenorphine program	Sublingual buprenorphine prescribing infrastructure in place	
Organization culture	Welcoming of buprenorphine services	
	Leadership and staff committed to facilitating implementation efforts	
Culture of adopting new innovations (low-threshold clinic)	Sped up implementation time	
Outer Setting		
Barriers		How addressed
Specialty pharmacy requirement		Establishing relationship with helpful specialty pharmacy (for publicly insured patients)
Patient insurance plans		Addiction medicine fellow assistance with patients with private insurance or dually insured with Medicare/Medicaid
		Support staff providing one-on-one help to participants to call Medicaid plans for reactivation
Facilitators		How leveraged
NYS Medicaid coverage of LAIB		Lack of prior authorization requirement allowed for timely receipt of medication from specialty pharmacy

**CFIR**: Consolidated Framework for Implementation Research; **DEA**: Drug Enforcement Administration; **EHR**: electronic health record; **LAIB**: long-acting injectable buprenorphine; **NYS**: New York State; **sublingual buprenorphine**: sublingual buprenorphine; **SSP**: syringe services program

## Data Availability

The datasets used and/or analysed during the current study are available from the corresponding author on reasonable request.

## Box 1. Implementation strategies

- Clinical champions
- Clinical working groups
- Facilitation
- Developing patient-facing handouts
- Developing clinical protocols
- Electronic health record smart phrases
- Staff trainings

## ABBREVIATIONS

CFIR: Consolidated Framework for Implementation Research
DEA: Drug Enforcement Administration
ECHO: Extension for Community Healthcare Outcomes
EHR: Electronic Health Record
ERIC: Expert Recommendations for Implementing Change
FDA: Food and Drug Administration
FQHC: Federally Qualified Health Center
HIV: Human Immunodeficiency Virus
LAIB: Long–Acting Injectable Buprenorphine
LPN: Licensed Practical Nurse
MCO: Medicaid Managed Care Organization
MOUD: Medications for Opioid Use Disorder
OUD: Opioid Use Disorder
PCSS: Providers Clinical Support System
PI: Principal Investigator
RN: Registered Nurse
SSP: Syringe Services Program
